# Prevalence and Risk Factors of Bovine and Ovine Lungworm Infection at Durame District, Southern Ethiopia

**DOI:** 10.1155/2021/6637718

**Published:** 2021-12-02

**Authors:** Haben Fesseha, Mesfin Mathewos

**Affiliations:** School of Veterinary Medicine, Wolaita Sodo University, P. O. Box, 138 Wolaita Sodo, Ethiopia

## Abstract

**Background:**

Lungworms are parasitic nematodes of the order Strongylidae that infect the lungs of cattle and sheep and cause bronchitis or pneumonia.

**Methods:**

An abattoir-based cross-sectional study was conducted from November 2018 to April 2019 to determine the comparative prevalence and the possible risk factors of lungworm infection in sheep and cattle of Durame district of Kembata Tembaro zone. For this purpose, a total of 410 animals (209 sheep and 201 cattle) were randomly selected and examined for the presence of different lungworm species using the modified Baermann technique and postmortem examination. The sensitivity and specificity of fecal sample test considering necropsy as reference/gold standard was conducted.

**Results:**

Accordingly, the overall prevalence of lungworm infection in sheep and cattle was 24.39%. *Dictyocaulus filaria* and *D. viviparous* were the only species of lungworm identified in sheep and cattle with a respective prevalence of 44.02% and 3.98%. Putative risk factors such as species and poor body condition have a significant association (*p* < 0.05) with the occurrence of lungworms in sheep and cattle. The lungworm infection was higher in the young age group (25.30%) and poor body conditioned (32.14%) animals. Season-wise prevalence revealed that a higher lungworm infection was recorded during autumn (32.14%) as compared to winter (22.81%) and spring (16.2%). Most of the sheep and cattle in the current study were heavily (45.0%) and moderately (39.0%) infected with lungworms whereas the rest 16.0% were infected with a low degree of lungworm infection. The specificity, sensitivity, PPV, and NPV of the modified Baermann technique against the gold standard test were 89.4%, 42%, 56.0%, and 82.7%, respectively.

**Conclusion:**

The study revealed that lungworm infection is prevalent in sheep and cattle of the study area and that was commonly occurs during autumn and affects poorly conditioned sheep and cattle. Thus, routine and strategic deworming for the control and prevention of lungworms in domestic animals should be recommended to overwhelm the prevalence of lungworm infection.

## 1. Introduction

Ethiopia is one of the developing countries owning of largest livestock population in Africa [1] with more than 85% of its population engaged in farming activities and contributing 40 percent to the total GDP [2, 3]. Ethiopia holds the largest livestock population in Africa with approximately 59.5 million cattle, 30.7 million sheep, 30.2 million goats, 4.5 million donkeys, 1.7 million horses, 0.33 million mules, 56.53 million poultry, and 4.9 million beehives [4]. Livestock serves for Ethiopian economy as sources of food, traction, manure, raw materials, investment, cash income, security, foreign exchange earnings, and social and cultural identity [2, 5].

The contributions of cattle and sheep, however, are overwhelmed due to several parasitic diseases that are responsible for different types of respiratory diseases. Lungworms are recognized as one of the major and prevalent parasitic diseases of ruminants worldwide responsible for bronchitis and pneumonia [6–8]. Lungworms are nematode parasites that are widely distributed throughout the world but are particularly common in countries with temperate climates and the highlands of tropical and subtropical countries and are widespread in Ethiopia [7, 9]. The species of importance in ruminants belong to two different families; the Dictyocaulidae and the Metastrongylidae [10]. The Dictyocaulidae include *Dictyocaulus viviparus* in cattle and buffaloes and *Dictyocaulus filaria* in sheep and goats [11, 12]. This parasite causes a severe pulmonary disease commonly called parasitic bronchitis, dictyocaulosis, or husk [13].

Affected herds usually show a high disease prevalence and mortality depending on the degree of pasture contamination [14] which is associated with rainfall that stimulates the activity of larvae [15]. Besides, an area's climatic condition and the presence of favorable environmental conditions for the larvae are also important predisposing factors for the prevalence of lungworm infection [16]. These two species of *Dictyocaulus* have a direct life cycle and those adult females lay larvated eggs in the bronchi. The eggs are coughed up and swallowed with mucus and the L_1_ hatch is excreted in feces during their passage through the gastrointestinal tract and L_1_. The larvae molt into the second stage (L_2_) on pasture and grow into the infectious L_3_. Then, the animal ingests it while grazing in the pasture [17].

Clinically, lungworm infection ranges from mild coughing with slightly higher respiratory rates to extreme persistent coughing (verminous pneumonia). In the case of associated bronchopneumonia, unthriftiness, dyspnea, nasal discharge, weight loss, fever, and death are major clinical signs [18]. The diagnosis may be focused on clinical symptoms and the history of grazing. The clinical signs, the time of the year, and the history of grazing are generally enough to make a diagnosis [19]. Lungworm is verified by the Baermann method by detecting the L_1_ stage in fecal samples [20].

Lungworm control and prevention can be accomplished by deworming all animals at the end of the rainy season to avoid heavy parasitic burden during grazing and deworming all animals before the rain begins at the end of the dry season, as it is very important to minimize pollution of pasture [12]. It is very important to provide balanced nutrition to keep animals safe and help them develop sufficient resistance to external pathogens [21]. The other solution is a vaccine that was produced from the *Dictyocaulus* larvae [22].

In Ethiopia, several researchers have documented the prevalence of lungworms in ruminants and the parasite species involved [16, 23–30]. However, only a few studies are available on bovine lungworms, in comparison to the existence of many studies on ovine lungworms. Studies that produce basic information on the prevalence, species diversity, and risk factors are extremely helpful for developing effective control and prevention measures in Ethiopia. Therefore, this study was conducted to compare the prevalence and assess the possible risk factors of lungworm infection in sheep and cattle in the Durame district of Southern Ethiopia.

## 2. Material and Methods

### 2.1. Study Area

This study was conducted in the municipal abattoir of Durame district which is an administrative center of the Kembata Tembaro Zone of the Southern Nations, Nationalities, and Peoples Region. The town has a latitude and longitude of 7°14′N 37°53′E with an elevation of 2101 meters above sea level. The zone possesses 7 woredas, 120 rural and 22 urban kebeles (smallest administrative unit in Ethiopia), and 14,1952 hectares of land. It has a total human population of 828,002. About 14.3% of the population live in urban while the remaining 85.7% are rural dwellers. Kembata Tambaro zone is gifted with enormous livestock resources and livestock contribute to household livelihoods through a variety of direct and indirect ways. The estimated livestock population of the zone is 169,265 cattle (composed of 140,432 local Zebus and 28,833 crossbred), 250,736 sheep and goats, 61,133 equine, 339,712 poultry, and 34,095 bees [31].

### 2.2. Study Animals

The study animals were indigenous cattle and sheep of various body condition scores, both sexes, and age groups and were presented to municipal abattoirs of Durame district for slaughter. The age of both cattle and sheep was estimated based on owners information and examining the dentition patterns according to the recommendation given by De Lahunta and Habel [32] and grouped into young (<6 years) and adult (6 years) age groups (cattle) whereas the age of the sheep was classified into young (0 to 1 year), adult (2 to 4 years), and old (above 4 years old animal) as per the recommendation given by Gatenby [33]. The body condition score of cattle was given according to Nicholson and Butterworth [34] with a scale of 1.0 to 9.0 and categorized into good (7 was classified as good/fat), medium (4, 5, 6, were medium), and poor (2, 3 were categorized as poor/lean) body conditions whereas the scoring of the body condition of the goats was determined according to Villaquiran et al. [35] with a scale of 1.0 to 5.0. Thus, goats with a score of 1 and 2 were categorized as poor, 3 as a medium, and 4 and 5 as good.

### 2.3. Study Design

A cross-sectional study was carried out from November 2018 to April 2019 to compare the prevalence and assess the possible risk factors of lungworm infection in sheep and cattle in the study area.

### 2.4. Sampling Method and Sample Size Determination

The simple random technique was employed to select study animals from the municipal abattoir of the Durame district. The random selection of study animals for fecal sampling was determined based total number of animals slaughtered per each abattoir visit. A perusal of different previous researches, no study has been done so far on comparative prevalence of lungworm infection of sheep and cattle in Durame district. Thus, the sample size was determined using the formula described by Thrusfield, [36], 50% expected prevalence and 5% desired absolute precision at a 95% confidence level.

(1) $$\begin{array}{c} n=\frac{1.96^{2}\text{Pexp }\left(1-\text{Pexp}\right)}{d^{2}}, \end{array}$$

where *n* is the required sample size, Pexp is the expected prevalence, *d* is the desired absolute precision, and 1.96^2^ is the *z* value for 95% confidence interval. Thus, the number of animals needed in this study was 384. However; to increase the level of precision, the study sample size has been increased to 410 (209 sheep and 201 cattle).

### 2.5. Sample Collection and Examination

After proper restraining, fecal samples were collected directly from the rectum and freshly dropped faces using disposable gloves. These samples were collected at the abattoir during the antemortem inspection. Also, lungworm larvae were collected by screw-capped universal bottle from the municipal abattoir of the study area via making an incision on the trachea down to bronchi and bronchioles during postmortem inspection. The samples were placed in an icebox and transported to Sodo regional veterinary laboratory and processed parasitologically.

#### 2.5.1. Modified Baermann Technique

In the laboratory, 25 grams of fresh faeces were weighed from each faecal sample for the recovery of L_1_ larvae using the modified Baermann technique. All the fecal examination was conducted according to the standard protocols [37]. The preservative was enclosed in gauze fixed on a string rod and submerged in a clean glass tube filled with fresh water and left for 24 hours. The larvae left the feces and migrate through the gauzes and settle at the bottom of the glass. After the drain off of the supernatant, the sediment was examined by stereomicroscope to detect the presence or absence of larvae [38–40]. When positive, a drop of 1% iodine solution was used to immobilize the larvae for the identification of species using a compound microscope. The larval identification of ruminant lungworms was then taken place based on their morphological characteristics. Accordingly, *D. filaria* was identified by its protoplasmic knob on its head and *D. viviparous* without a protoplasmic knob on its head [41].

#### 2.5.2. Postmortem Examination

Each of the study animals was given an identification number with a paint mark on their body during the antemortem examination. Using a data collection format, comprehensive records of the species, age, sex, and body conditions of the study animals were carried out. During the postmortem examination, the lungs were first examined by visual inspection and palpation, and then, an incision was made to assess the presence, size (load), and species of lungworm [42]. Each lung from the study animals was inspected by incising it starting from the trachea down to bronchi and bronchioles and then making multiple deep incisions of the lobes with several small subcuts. The recovered worms were kept in 70% alcohol and then were transferred to the laboratory for examination. Adult parasites were examined under the stereomicroscope for determining the degree of lungworm parasitic burden; [43, 44] and the identification (classification) of adult lungworm parasites to the species level as has been done previously [45].

### 2.6. Data Management and Statistical Analysis

The data collected was entered into the Microsoft Excel 2019 Spreadsheet and analyzed using STATA version 13. Descriptive statistics such as percentages were used to label the prevalence of lungworm infection. Logistic regression analyses were conducted using lungworm infection as outcome variables against each of the explanatory variables of the hypothesized risk factors (animal species, sex, age, body condition, and season of the year). The explanatory variables with a *p* value ≤ 0.25 in univariable analyses were selected for multiple logistic regression analyses. The final multiple logistic regression models were manually built using a forward stepwise selection approach. A variable was to be considered a confounder if it changed the coefficient of the significant variables by more than 25%. Multicollinearity of the predictors in the models was also assessed using Kruskal gamma statistics, and those variables whose gamma value ranged between -0.6 and +0.6 were considered in a multivariable logistic regression model. The odds ratio (OR) and its 95% confidence interval (CI) of the variables associated with the outcome variables were calculated from the final multivariate logistic regression models. Considering the necropsy finding of lungworm as a “gold” standard diagnostic test; sensitivity and specificity of the modified Baermann technique were calculated against the gold standard test. Levels of significant differences were considered at a *p* value less than 0.05. The method of calculating sensitivity, specificity, positive predictive value (PPV), and negative predictive value (NPV) of the Baermann technique was described in Table 1.

### 2.7. Ethical Clearance

The best practice guidelines for veterinary care were followed and those cattle and sheep owners were informed as to the purpose of the study and that the Wolaita Sodo University of Research Ethics and Review Committee approved the verbally informed consent process in the manuscript.

## 3. Results

### 3.1. Comparative Prevalence of Lungworm Infection in Cattle and Sheep

From a total of 410 fecal samples examined, 100 was positive for one or more eggs of lungworms of sheep and cattle and revealed an overall prevalence of 24.39%. There was also a significant variation (*p* ≤ 0.001) between lungworm infection of the two species. The prevalence of lungworm infection in sheep and cattle was 44.02% and 3.98%, respectively (Table 2).

### 3.2. Frequency of Parasitic Burden in Cattle and Sheep

Out of the total of 100 infected animals, about 45.0% and 39.0% of the animals were heavily and moderately infected with lungworms, respectively, whereas lower lungworm infection rates were recorded in 16.0% of the study animals (Table 3).

### 3.3. Factors That Contribute to the Prevalence of Lungworm Infection in Cattle and Sheep

The result of univariable and multivariate logistic regression analyses of the prevalence of lungworm with various risk factors has been shown in Table 4. Factors such as species, body condition, and season were considered in the study since they were found significant (*p* < 0.05) whereas age and sex were excluded (*p* > 0.25). All those independent variables, which were significant in the initial univariable analysis checked for collinearity using Kruskal gamma statistics, and those variables whose gamma value ranged between −0.6 and +0.6 were considered in a multivariable logistic regression model. Accordingly, no collinearity was detected between these variables and was used for multivariable analysis. Thus, species, body condition, and season were included in the multivariable model; however, only species was found significantly (*p* < 0.05) associated with lungworm (Table 4). The Hosmer-Lemeshow goodness of fit test suggested that the model fit the data (*χ*^2^ = 10.55; *p* = 0.56; ROC = 82.3%).

In the present study, a higher prevalence of lungworms was recorded in males (25.78%), young (25.3%), poorly conditioned animals (32.14%), and autumn season (32.14%) (Table 4).

### 3.4. Performance of the Laboratory Techniques

Considering necropsy as the gold standard test, the specificity, sensitivity, PPV, and NPV of the modified Baermann technique were 89.4%, 42%, 56.0%, and 82.7%, respectively. The type of laboratory technique used has a significant (*χ*^2^ = 67; *p* = 0.0001) effect on the determination of the prevalence of lungworm (Table 5).

## 4. Discussion

The results of the present study revealed a high infection of lungworms in cattle and sheep that was presented to the municipal abattoir of Durame district and revealed an overall prevalence of 24.39%. The prevalence of lungworm in sheep and cattle was 44.02% and 3.98%, respectively, and a statistically significant association (*p* < 0.05) was found between the species of animal and lungworm infection. The prevalence of bovine lungworm in the present study was higher than the previous study of Yildiz [46] who reported zero prevalence in the Kirikkale province of Turkey, Fekadu [47] who reported 0.5% in the Addis Ababa abattoir, and Wolde and Mersha [29] who reported 1.5% in Addis Ababa abattoir, while the current prevalence was comparable with the finding of Menzir and Dessie [26] (3.1%) in Gondar. On the contrary, our finding was lower than the report of Yihenew and Temesgen [30] who reported a 10% prevalence in Kembibit District, Central Ethiopia. The variation in the prevalence of lungworms in cattle can be linked to the farmer's treatment practice of their animals, especially those cattle kept feedlot and later brought to slaughter which may result in low disease exposure [48, 49]. Furthermore, the owners kept cattle for fattening normally treat with broad-spectrum anthelmintics that might significantly variation and reduce lungworm prevalence.

The overall prevalence of lungworm in sheep was 44.02% was higher than the finding of Gebreyohannes et al. [50] 8.6% in Mekedella district, Muluken [51] 18.2%, and Kassa and Abdu [52] 20.2% in Bahir Dar district. Our finding was comparable with the earlier reports of Basaznew et al. [53] 43.3% in Dessie Zuriya, Terefe et al. [54] 42.0% in North Gondar Zone, and Mezgebu [55] 48% in Addis Ababa. However, the results of the present study were higher than the reports of Yosef and Solomon [25] 56.3% in Debre Birhan district, Terefe [28] in the Dessie area (60.5%), and Kombolcha (67.8%), and Paulos [56] 81.0% in the Chilallo area. Such variation in the prevalence of lungworms in sheep could be attributed to the difference in study type, sample size, study area, and month and seasons of the year of sample collections by different studies.

The species of lungworms identified in the current study were *Dictyocaulus filaria* and *Dictyocaulus viviparous*. The presence of these lungworms in the present study area could be associated with their life cycle as they have a direct life cycle that did not require a molluscan intermediate host to complete their development and the season of the study. This finding concurred with previous reports of Rahmeto et al. [9], Fentahun et al. [27], Moges et al. [57], Addis et al. [58], and Kebede et al. [16] who reported D*. filaria* and *D. viviparous* were the most predominant species circulating in Ethiopian sheep and cattle managed under traditional husbandry system.

The distribution of lungworm occurrences according to the season of the year revealed that the peak lungworm load was observed during autumn (32.14%) and becomes diminution to a minimum during spring (16.2%). Therefore, seasonal dynamics influence the occurrence of lungworms in sheep and cattle. Our findings disagreed with the previous reports by other researchers [15, 23, 24, 59] who reported higher prevalence during spring and summer. The finding of a heavy degree of infection during the autumn and winter while minimal heavy parasitic burden during the spring was due to the highest availability of active infective lungworm larvae in autumn and winter as the climatic condition favors them. This allowed sheep to ingest a large number of infective larvae. Late summer/autumn is the key risk period for young animals so treat at intervals during this time, according to the anthelmintic used [15, 16].

The overall prevalence of the degree of lungworm infection in both sheep and cattle was represented by 45.0%, 39.0%, and 16% for heavy, moderate, and low parasitic infections, respectively. This could be due to an increase in the degree of pasture contamination in an extensive system of production may increase the degree of exposure, so result in a high degree of parasitic burden [8]. Also, the reason for this could partly be since poorly nourished animals were more prone to lungworm infection, and although it is not usual for well-fed animals to succumb to the disease if a conducive environment were made [60].

## 5. Conclusion

This study revealed that a higher prevalence of lungworm infection in sheep and cattle. *D. filaria* and *D. viviparous* are the dominant lungworm species in the study area. A higher prevalence of lungworm infection was noted during autumn as compared to winter and spring. Thus, studies are needed to clarify issues regarding seasonal variations of lungworms in all domestic animals to with awareness creation to the society on the prohibition of animals from grazing early in the morning and evening when there is high activity of larvae due to moisture when pasture contamination is very high. Besides, strategic anthelmintic drug treatment or deworming should be implemented at the beginning of the rainy season and the end of the rainy season could appear to be most effective.

## Figures and Tables

**Table 1:** Method of calculating sensitivity, specificity, PPV, and NPV.

Techniques	Gold standard (necropsy)		
Modified Baermann	Positive	Negative	Total
Positive	a	b	a/a+b (PPV)
Negative	c	d	d/c+d (NPV)
Total	a+c	b+d	
	a/a+c (sensitivity)	d/b+d (specificity)	

**Table 2:** Comparative proportion of lungworm infection in sheep and cattle.

Species	No. of examined animal	No. of positive animals	Prevalence (%)	Chi-square (*χ*^2^)	*p* value
Cattle	201	8	3.98	89.07	<0.001
Sheep	209	92	44.02		
Total	410	100	24.39		

**Table 3:** Proportion of the degree of lungworm infection.

Degree of lungworm infection	No. of positives	Proportion (%)
Low	16	16.0
Moderate	39	39.0
Heavy	45	45.0

**Table 4:** Univariate and multivariate logistic regression analyses of risk factors for bovine and ovine lungworms infection.

Risk factors	No. of examined animal	No. of positive animals	Binary logistic regression analysis		Multivariate logistic regression analysis		
			COR (95% CI)	*p* value	AOR (95% CI)	*p* value
Species						
Cattle	201	8 (3.98)	Ref	Ref	Ref	Ref
Sheep	209	92 (44.02)	18.97 (8.89-40.49)	<0.001	19.7 (9.16-42.41)	<0.001
Sex						
Female	185	4222.70	Ref	Ref	—	—
Male	225	5825.78	1.18 (0.75-1.86)	0.471	—	—
Age						
Adult	244	58 (23.78)	Ref	Ref	—	—
Young	166	42 (25.30)	1.09 (0.58-1.45)	0.723	—	—
BCS						
Good	99	16 (16.2)	Ref	Ref	Ref	Ref
Medium	171	39 (22.81)	1.53 (0.81-2.92)	0.193	1.3 (0.62-2.70)	0.483
Poor	140	45 (32.14)	2.46 (1.29-4.67)	0.006	2.0 (0.95-4.23)	0.069
Season						
Autumn	171	39 (22.81)	1.60 (0.97-2.65)	0.066	1.44 (0.77-2.68)	0.252
Spring	140	45 (32.14)	0.65 (0.34-1.24)	0.193	0.63 (0.30-1.30)	0.212
Winter	99	16 (16.2)	Ref	Ref	Ref	Ref

**Table 5:** Sensitivity and specificity of diagnostic techniques against the gold standard method.

Techniques	Gold standard (necropsy)					
	Sensitivity	Specificity	PPV	NPV	*p*, *χ*^2^	Kappa value
Modified Baermann	42.0%	89.4%	56.0%	82.7%	67 (0.0001	0.243

## Data Availability

The data used to support the findings of this study are available from the corresponding author upon request.

## Abbreviations

AOR: Adjusted odds ratio
BCS: Body condition score
CI: Confidence interval
COR: Crude odds ratio
PPV: Positive predictive value
NPV: Negative predictive value.
